# The Involvement of the *S2P2* Intramembrane Protease in the Response of *Arabidopsis thaliana* Chloroplasts to High Light Stress

**DOI:** 10.3390/plants14162584

**Published:** 2025-08-20

**Authors:** Maria Ciesielska, Małgorzata Adamiec, Robert Luciński

**Affiliations:** Department of Plant Physiology, Institute of Experimental Biology, Faculty of Biology, Adam Mickiewicz University in Poznań, ul Uniwersytetu Poznańskiego 6, 61-614 Poznań, Poland; maria.ciesielska1604@gmail.com (M.C.); msolin@amu.edu.pl (M.A.)

**Keywords:** high irradiance, intramembrane protease, photosystem II, photosynthesis, proteolysis, thylakoid

## Abstract

High light intensity constitutes a critical abiotic stress factor that profoundly affects the structural and functional integrity of the photosynthetic apparatus. Excessive irradiance triggers accelerated degradation of the PsbA polypeptide, increases susceptibility to photoinhibition, and promotes overproduction of reactive oxygen species (ROS), thereby inducing oxidative damage to proteins, lipids, and nucleic acids. Among the chloroplast-localized site-2 proteases of *Arabidopsis thaliana*, *S2P2* remains the least characterized. In this study, our analyses revealed a pronounced upregulation of the *S2P2* (AT1G05140) gene and a concomitant accumulation of the *S2P2* protein under high light conditions. Functional characterization using two independent *S2P2* insertional mutant lines lacking the protease demonstrated that loss of *S2P2* significantly exacerbates photoinhibition. Mutants exhibited reduced photosystem II (PSII) efficiency, accompanied by accelerated degradation of the PSII core proteins PsbA, PsbD, and PsbC, as well as elevated ROS generation. These findings provide the first direct evidence that *S2P2* plays a pivotal role in maintaining the stoichiometric balance of PSII core components and conferring resilience of the photosynthetic machinery to high light stress. This work expands the functional repertoire of chloroplast site-2 proteases and underscores *S2P2* as a potential target for improving stress tolerance in plants.

## 1. Introduction

The acclimation of plants to constantly changing environmental conditions requires the involvement of numerous precise regulatory mechanisms. One of the most fundamental among them is proteolysis. Proteases are involved, among other functions, in protein quality control and protein turnover processes. These mechanisms are relatively well understood and have been extensively described in the literature [1,2,3,4,5]. However, in recent years, a surprising novel process related to proteolytic activity has been discovered—regulated intramembrane proteolysis (RIP). This process is carried out by a highly specific group of proteolytic enzymes known as intramembrane proteases. These enzymes are transmembrane proteins that exhibit protease activity within the lipid bilayer of biological membranes. They are widely recognized for their role in the cleavage and release of membrane-anchored transcription factors from biological membranes [6,7,8,9]. To date, numerous enzymes belonging to various classes of intramembrane proteases have been identified (for details, see [9]).

In plant cells, the first identified intramembrane protease was the chloroplast-localized protein EGY1 (ethylene-dependent gravitropism deficient and yellow-green 1), which belongs to the site-2 protease (S2P) family [10]. The absence of this protease in *A. thaliana* cells results in numerous pleiotropic effects, the most characteristic of which are yellow-green leaf pigmentation and a deficiency in ethylene-dependent gravitropism. EGY1-deficient mutants exhibit a poorly developed internal membrane [10,11]. Moreover, EGY1 has been shown to participate in plant responses to ammonium and phosphate stresses [12,13].

In addition to EGY1, other S2Ps are also localized in the chloroplasts of *A. thaliana*, namely EGY2, AraSP, and *S2P2* [9]. EGY3, which is also present in the thylakoid membranes of chloroplasts, is closely related to EGY1 and EGY2; however, due to the absence of a key motif required for proteolytic activity, it is proteolytically inactive and is classified as a so-called pseudo-protease [9,14,15,16]. Within chloroplasts, all of the mentioned proteases—except AraSP—are localized in the thylakoid membranes [9,14,15,17]. AraSP is the only one found in the chloroplast envelope membranes [18].

The physiological roles and precise molecular mechanisms of action of S2Ps remain poorly understood. It has been shown that AraSP plays an essential role in chloroplast biogenesis and plant development. *A. thaliana* mutants lacking this protease are characterized by extremely small size, poorly developed root systems, reddish cotyledons, and a drastically shortened lifespan of less than 20 days [18]. However, the exact molecular mechanisms underlying these phenotypes have yet to be elucidated.

Similarly, *A. thaliana* mutants deficient in EGY2 display a number of phenotypic abnormalities, including shortened hypocotyls, reduced fatty acid content, and increased sensitivity to photoinhibition [14,19,20].

To date, virtually nothing is known about the protein substrates of chloroplast-localized S2Ps. Only our previous studies have indicated that the transcription factor pTAC16, which is also localized in the thylakoid membranes and is involved in interactions with the plastid-encoded RNA polymerase complex, may represent a natural substrate of EGY2 [20].

A substantial body of literature indicates the involvement of chloroplast-localized S2Ps—particularly EGY1 and EGY2—in the regulation of stoichiometric relationships between the polypeptides that constitute photosystem II (PSII) and its major light-harvesting antenna complex, LHCII [10,13,19,20,21,22,23]. The absence of either EGY1 or EGY2 in *A. thaliana* mutant cells is also associated with increased plant sensitivity to photoinhibition [19,20,23].

Among the chloroplast S2Ps, *S2P2* remains the least characterized. The *S2P2* (AT1G05140) gene exhibits a high level of expression in both cotyledons and mature leaves of *A. thaliana*, suggesting a potentially important role in chloroplast development and function [24]. *S2P2* has also been proposed to participate in the plant’s response to cold stress [25]. Interestingly, until recently, the intrachloroplast localization of *S2P2* remained unknown. It was only through our recent study that *S2P2* was definitively localized to the thylakoid membranes, similarly to EGY1, EGY2, and the pseudo-protease EGY3 [16].

Moreover, we demonstrated that the loss of *S2P2* in *A. thaliana* leads to disruptions in chloroplast pigment content and reductions in the levels of core PSII polypeptides and LHCII trimers. *A. thaliana* mutants lacking *S2P2* showed decreased levels of PsbA, PsbC, and PsbD proteins, as well as the light-harvesting proteins Lhcb1 and Lhcb2. These changes were accompanied by a reduced abundance of PSII–LHCII supercomplexes under light-optimal conditions and a marked increase in sensitivity to photoinhibition [16].

Considering this information, we decided to investigate the involvement of the *S2P2* protease in the response of *A. thaliana* chloroplasts to high light (HL) stress. Specifically, we examined how the levels of key core PSII proteins—PsbA, PsbC, and PsbD—are affected during prolonged high light exposure, and how these changes influence PSII function. Our results showed that *S2P2* gene expression is significantly induced in response to high light intensity, accompanied by an increase in the abundance of the *S2P2* protease.

Stress conditions are often associated with a marked increase in the accumulation of reactive oxygen species (ROS) within plant cells. According to the literature [26] and our previous work [19,22], *egy2* mutants exhibit significantly elevated ROS levels under stress conditions compared to wild-type (WT) plants. Based on the fact that *S2P2* also belongs to the chloroplast S2Ps, we decided to verify the hypothesis regarding the generation of increased amounts of ROS under high light stress conditions in *S2P2* mutants. This is the first report demonstrating that the absence of the *S2P2* protease leads to a substantial impairment of the photosynthetic apparatus in *A. thaliana* and causes excessive ROS production under high light conditions. These findings identify *S2P2* as an important additional component of the complex regulatory network responsible for chloroplast acclimation to fluctuating light environments.

## 2. Results

### 2.1. Changes in S2P2 Gene Expression and Protein Abundance in Response to High Light Stress in A. thaliana Leaves

**Figure 1:**
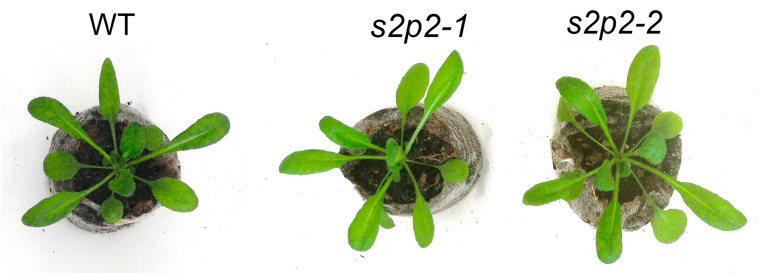
The 4-week old WT, *s2p2–1*, and *s2p2–2* mutant line plants.

Quantitative real-time PCR (qRT-PCR) was employed to assess the expression dynamics of the *S2P2* gene in response to high light stress. Four-week-old *A. thaliana* WT plants were exposed to high light intensity (1000 μmol m^−2^ s^−1^) for 0, 3, 6, 12, and 24 h (Figure 1). 

**Figure 2:**
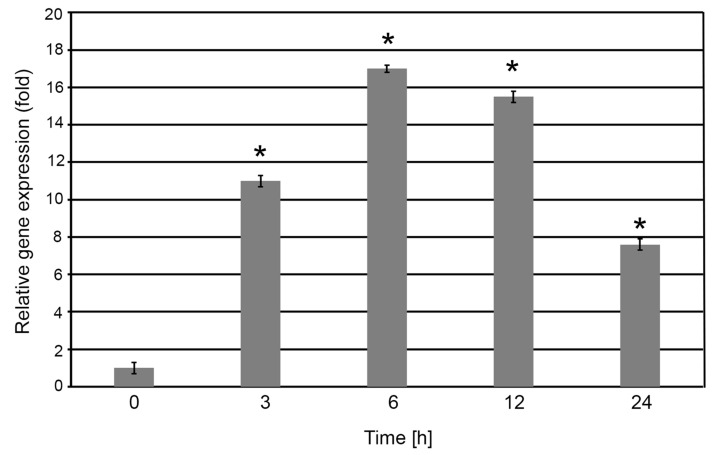
Relative expression of the *S2P2* gene in *A. thaliana* leaves exposed to high light conditions. Four-week-old wild-type *A. thaliana* plants were subjected to high light intensity (1000 μmol m^−2^ s^−1^) for 0, 3, 6, 12, and 24 h. Following treatment, total RNA was extracted from leaf tissues and reverse-transcribed into complementary DNA (cDNA), which was subsequently used as a template for quantitative real-time PCR (qRT-PCR) analysis. Expression level of *S2P2* was normalized against the endogenous reference gene *CYP5*. Asterisks (*) indicate statistically significant changes compared to the 0 h base time value (*p* < 0.05).

A rapid and significant upregulation of *S2P2* transcript levels was observed as early as 3 h post-exposure, reaching approximately an 11-fold increase relative to the control (0 h). Between 3 and 6 h, *S2P2* expression increased by an additional ~30%. This elevated expression level remained relatively stable up to 12 h. After 24 h of high light exposure, *S2P2* transcript levels declined by approximately 50% compared to the peak level but remained above the baseline expression level observed under control light conditions (Figure 2).

**Figure 3:**
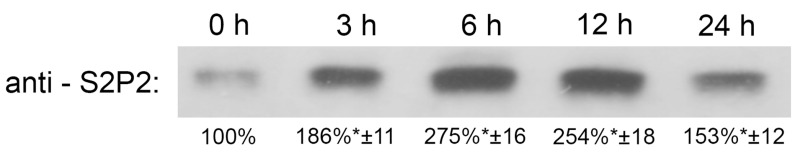
Immunoblot-based quantification of *S2P2* protein levels in wild-type *A. thaliana* exposed to high light. Wild-type *A. thaliana* plants were exposed to high light intensity (1000 μmol m^−2^ s^−1^) for 0, 3, 6, 12, and 24 hours. Protein extracts from leaf tissues were subjected to immunoblot analysis to determine *S2P2* protein abundance. Protein concentration in extracts was determined according to the Lowry method. Before hybridization with antibodies, the membranes were transiently stained with Ponceu S to verify the transfer efficiency and control loading. The experiment was conducted using three independent biological replicates. Data are presented as mean ± standard deviation (s.d.). Asterisks (*) denote statistically significant differences related to the 0 h time point (*p* < 0.05).

The transcriptional activation of *S2P2* was accompanied by a corresponding increase in *S2P2* protein accumulation. Western blot analysis revealed that *S2P2* protein levels increased to over 180% of the initial level after 3 h of high light treatment. Continued exposure led to further accumulation, reaching approximately 275% of the control level at 6 h. Similar to transcript levels, *S2P2* protein abundance began to decline by 24 h, although it remained elevated compared to the control (Figure 3). These results indicate a strong correlation between *S2P2* gene expression and protein accumulation in response to high light stress.

### 2.2. Functional Status of Photosystem II in S2P2 Mutant Lines Under High Light Stress

To assess whether the loss of *S2P2* protease function affects PSII performance during the exposure of plants to excessive irradiance, we evaluated PSII activity in two independent *S2P2* mutant lines using pulse amplitude-modulated (PAM) chlorophyll fluorescence analysis. The parameters measured included minimum fluorescence yield (F_0_), maximum quantum efficiency of PSII photochemistry (F_v_/F_m_), photochemical quenching (qP), and non-photochemical quenching (NPQ).

**Figure 4:**
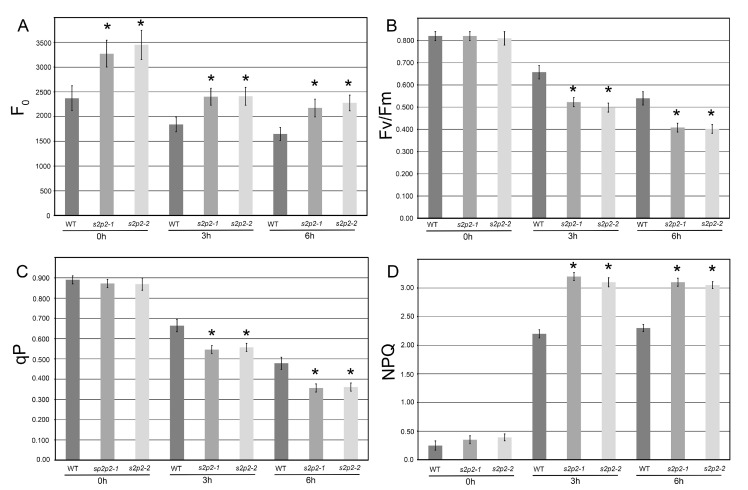
Chlorophyll *a* fluorescence parameters in wild-type and *S2P2* mutant lines under high light stress. Chlorophyll *a* fluorescence measurements were conducted in wild-type *A. thaliana* (WT) plants and *s2p2–1* and *s2p2–2* mutant lines following exposure to high light intensity (1000 μmol m^−2^ s^−1^) for 0, 3, and 6 h. The following parameters were analyzed: (**A**) minimum fluorescence yield (F_0_), (**B**) maximum quantum efficiency of PSII (F_v_/F_m_), (**C**) photochemical quenching (qP), and (**D**) non-photochemical quenching (NPQ). Actinic light intensity matched the pre-dark adaptation irradiance: 110 μmol m^−2^ s^−1^ for control conditions (0 h) and 1000 μmol m^−2^ s^−1^ for high light stress treatments (3 h and 6 h). Measurements were performed on 30 individual plants per genotype across three independent biological replicates. Data are presented as mean ± standard deviation (s.d.). Asterisks (*) indicate statistically significant differences between WT and mutant lines (*p* ≤ 0.05).

Given that the most pronounced upregulation of *S2P2* gene expression occurred within the first 6 h of high light exposure, functional measurements were conducted after 3 and 6 h of treatment at high irradiance (1000 μmol m^−2^ s^−1^). Under control light conditions, both *S2P2* mutant lines exhibited elevated F_0_ levels compared to WT plants. During high light exposure, F_0_ levels remained higher in the mutants, although the relative decline in F_0_ appeared slightly more pronounced in *S2P2* mutant plants than in WT (Figure 4A).

Under standard light conditions, no significant differences were observed in qP, F_v_/F_m_, or NPQ between WT and mutant lines. However, following high light treatment, *S2P2* mutants showed a significantly greater reduction in both qP and F_v_/F_m_ values compared to WT plants, indicating a higher susceptibility of PSII to photoinhibition in the absence of functional *S2P2* intramembrane protease (Figure 4B,C).

The NPQ parameter, indicative of thermal energy dissipation, increased markedly in all genotypes under high light. Notably, the *S2P2* mutant lines exhibited a stronger NPQ response, with an average increase of approximately 40% compared to WT after 3 h of high light exposure (Figure 4D).

### 2.3. Abundance of Selected PSII Core Proteins in S2P2 Mutant Lines Under High Light Stress

Given the functional impairments in PSII observed in *S2P2* mutant lines under high light stress (Figure 4), we hypothesized that these mutants would also exhibit altered accumulation of PSII core proteins in comparison to WT plants. Therefore, we assessed the levels of three essential PSII core polypeptides—PsbA (D1), PsbD (D2), and PsbC (CP43)—in *S2P2* mutants during high light stress.

**Figure 5:**
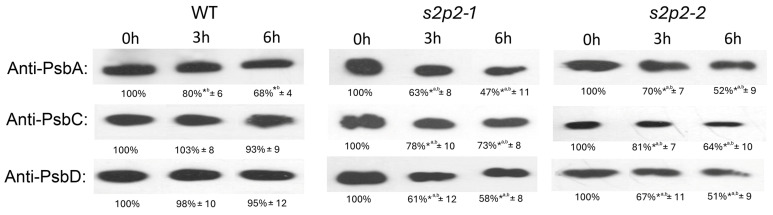
Immunoblot quantification of PsbA, PsbC, and PsbD proteins in wild-type and *S2P2* mutant lines under high light conditions. Protein levels of PsbA, PsbC, and PsbD were analyzed in wild-type *A. thaliana* (WT) plants and *s2p2–1* and *s2p2–2* mutant lines following exposure to high light intensity (1000 μmol m^−2^ s^−1^). Thylakoid membrane proteins (2 μg), isolated from leaf tissues, were separated by SDS-PAGE and subjected to immunoblot analysis using protein-specific primary antibodies. Protein concentration in extracts was determined according to the Lowry method. Before hybridization with antibodies the membranes were transiently stained with Ponceu S to verify the transfer efficiency and control loading. Quantification of immunoblot signals was performed using GelixOne software. Signal intensities were normalized to the corresponding 0 h time point (set as 100%) for each genotype. The experiment included three independent biological replicates. Data are expressed as mean ± standard deviation (s.d.). *^a^ denote statistically significant differences among treatments at identical time point; *^b^ indicate statistically significant differences related to 0 h time point in each genotype (*p* ≤ 0.05).

As expected, exposure to high light conditions resulted in a significant decrease in PsbA protein levels in WT *A. thaliana* plants. Both *s2p2–1* and *s2p2–2* mutant lines also exhibited a pronounced reduction in PsbA levels after 3 and 6 h of high light treatment. However, the rate of decline was notably faster in the mutant lines compared to WT (Figure 5).

In contrast, a different pattern was observed for PsbD and PsbC polypeptides. While their levels remained relatively stable in WT plants during the high light exposure, both *S2P2* mutant lines showed a marked reduction in the abundance of these proteins. After 6 h of stress, PsbD levels in *S2P2* mutants had declined to approximately 50% of the initial value, while PsbC content was reduced to 65–70% (Figure 5).

### 2.4. Accumulation of Superoxide Anion Radical and Hydrogen Peroxide in S2P2 Mutants Under High Light Stress

It is well established that high irradiance induces enhanced production of ROS in chloroplasts. These ROS are major contributors to oxidative stress, rapidly leading to oxidative damage of proteins, including those constituting photosynthetic complexes within thylakoid membranes. This oxidative damage is associated with a decline in the efficiency of photosynthetic reactions and reduced performance of the photosystems. The changes observed in PSII functionality and the reduced abundance of PSII core proteins in *S2P2* mutants under high light stress prompted us to examine ROS accumulation in the leaves of these mutants.

**Figure 6:**
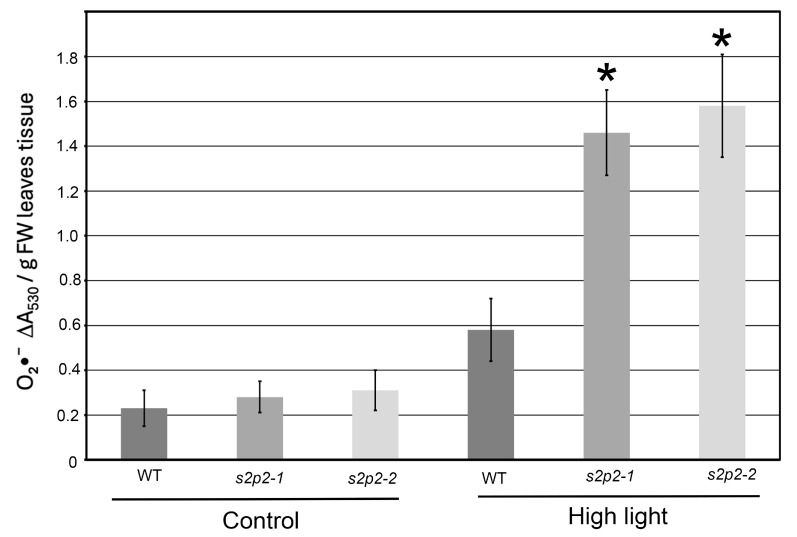
Superoxide radical accumulation in wild-type and *S2P2* mutant lines under high light stress. The accumulation of superoxide radicals (O_2_^•−^) was evaluated in wild-type *A. thaliana* (WT) plants and *s2p2–1* and *s2p2–2* mutant lines following exposure to high light intensity (1000 μmol m^−2^ s^−1^) based on the reduction in nitroblue tetrazolium (NBT). Quantitative data represent mean values from three independent biological replicates. Asterisks (*) indicate statistically significant differences between mutant lines and the wild-type plants (*p* ≤ 0.05).

**Figure 7:**
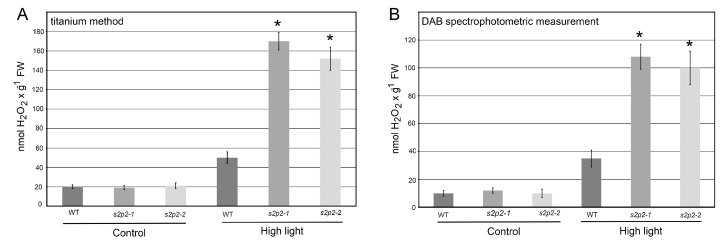
Hydrogen peroxide accumulation in wild-type and *S2P2* mutant lines following high light stress. Hydrogen peroxide (H_2_O_2_) accumulation was assessed in wild-type *A. thaliana* (WT) plants and *s2p2–1* and *s2p2–2* mutant lines after 3 h of exposure to high light conditions (1000 μmol m^−2^ s^−1^). (**A**) Titanium (Ti^4+^) staining method. (**B**) 3,3′-diaminobenzidine (DAB) staining spectrophotometric measurement. Results represent the mean values from three independent biological replicates, each comprising 30 plants. Asterisks (*) indicate statistically significant differences between mutant lines and the wild-type plants (*p* ≤ 0.05).

To this end, we quantified the levels of both superoxide radical (Figure 6) and hydrogen peroxide (Figure 7) in response to high light exposure. Hydrogen peroxide content was measured using two independent approaches: a titanium-based assay and spectrophotometric quantification using 3,3′-diaminobenzidine (DAB). Both methods yielded consistent results.

Under non-stress conditions, ROS levels in both WT and *S2P2* mutant leaves were low and comparable. However, following a 3 h exposure to high light intensity, ROS accumulation in the *S2P2* mutants significantly exceeded that in WT plants. Specifically, the level of superoxide radical in *S2P2* mutant leaves was over 60% higher than in WT leaves (Figure 6), while hydrogen peroxide content was more than threefold higher in the mutants compared to WT (Figure 7).

### 2.5. Changes in the Abundance of Copper/Zinc Superoxide Dismutase 2 (CSD2) During High Light Stress

Given the markedly elevated levels of both superoxide radical and hydrogen peroxide observed in the leaves of *S2P2* mutants under high light stress, we investigated the accumulation of CSD2 in chloroplasts under these conditions, as it is a key enzyme scavenging the superoxide radical generated in chloroplasts.

**Figure 8:**
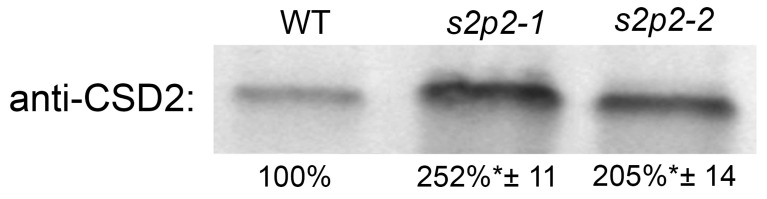
Immunoblot quantification of CSD2 protein in wild-type and *S2P2* mutant lines following high light exposure. CSD2 protein levels were analyzed in wild-type *A. thaliana* (WT) plants and *s2p2–1* and *s2p2–2* mutant lines after 3 h of exposure to high irradiance (1000 μmol m^−2^ s^−1^). Protein extracts from leaf tissues were subjected to SDS-PAGE and immunoblotting using a CSD2-specific primary antibody. Protein concentration in extracts was determined according to the Lowry method. Before hybridization with antibodies, the membranes were transiently stained with Ponceu S to verify the transfer efficiency and control loading. Quantification was based on three independent biological replicates. Data are presented as mean ± standard deviation (s.d.). Asterisks (*) indicate statistically significant differences between mutant lines and the WT control (*p* ≤ 0.05).

After 3 h of exposure to high light intensity, the abundance of the CSD2 protein in the chloroplasts of both the *s2p2–1* and *s2p2–2* mutant lines was more than twofold higher than that observed in the chloroplasts of WT plants (Figure 8). This elevated CSD2 level in the mutants likely represents a compensatory response to increased oxidative stress and enhanced superoxide radical production under high irradiance.

## 3. Discussion

*S2P2* is a nuclear-encoded member of the zinc metalloprotease family, specifically classified within the site-2 protease (S2P) family [9]. It is believed that intramembrane proteases such as *S2P2* function in regulated intramembrane proteolysis (RIP), a mechanism by which membrane-bound, protein transcription factors are cleaved and released to mediate changes in gene expression [9]. In *A. thaliana*, six genes encoding S2P family proteases have been identified: EGY1, EGY2, EGY3, S2P2, ARASP, and the product of At4g20310 [10,14,18,27,28]. With the exception of At4g20310, all are localized to the chloroplast. Much is already known about the significance and possible functions of EGY1 [10,13,15,21,22,23] and EGY2 proteases [14,19,20], as well as EGY3, which, due to the lack of the characteristic zinc-binding motif (HExxH), lacks proteolytic activity and is classified as a so-called pseudo-protease [16,29]. However, knowledge about the physiological functions of *S2P2* is extremely limited.

In our previous work [16], using a large set of immunological methods, we established the thylakoid membrane localization of the *S2P2* protease and highlighted its contribution to maintaining photosynthetic apparatus integrity under non-stressful conditions. In the present study, we investigated the role of *S2P2* in *A. thaliana* exposed to short-term high light stress. For most chloroplast S2Ps studied to date, their importance in maintaining the correct stoichiometry of PSII polypeptides under light stress has been observed. *A. thaliana* mutants lacking both EGY1 and EGY2 were characterized by significant changes in the abundance of key PSII core polypeptides under high light exposure. This resulted in the increased sensitivity of these mutants to photoinhibition, significantly less efficient PSII function, and reduced ability of the mutants to regenerate after light stress was removed [6,10,11,12,14,18,19,22].

However, no molecular mechanism leading to the effects observed in these mutants has been described to date. No endogenous substrates for S2P proteases in chloroplasts are known, with the exception of the transcription factor pTAC10, which we propose as a substrate for EGY2 [19].

We first examined the expression pattern of the *S2P2* gene and the corresponding protein abundance in WT plants subjected to high irradiance. A significant upregulation of *S2P2* transcripts was observed, which was strongly correlated with increased *S2P2* protein accumulation in chloroplasts. The relationship between protein accumulation and transcript levels depends, among other things, on the location and function of the protein [30,31]. Therefore, the observed increase in both *S2P2* transcript and protein levels strongly suggests that this protease plays a crucial role in modulating the chloroplast response to short-term high light stress.

It is well documented that high light intensity leads to the impairment of PSII function, which is associated with photoinhibition. The PSII core polypeptide PsbA, together with PsbD, forms the reaction center and is particularly vulnerable to photodamage [32,33,34]. Our previous studies have demonstrated that the absence of the *S2P2* intramembrane protease, as well as other chloroplast-localized S2Ps, can result in significant disruptions in the stoichiometry of core PSII proteins during non-stressful conditions [17,19,20,23]. Here, we investigated how the absence of the *S2P2* protease affects the behavior of *A. thaliana S2P2* mutants exposed to short-term light stress. Based on our previous studies [4,18] and the existing literature [22,35], it is well established that short-term exposure (3–6 h) to high light intensity (1000 μmol m^−2^ s^−1^) is sufficient to induce measurable changes in the abundance of proteins constituting the PSII complex.

Western blot analyses revealed that the absence of *S2P2* resulted in a more pronounced degradation of PsbA, PsbD, and PsbC polypeptides under high light stress in both the *s2p2–1* and *s2p2–2* mutant lines compared to WT. The strong degradation of PsbA in both WT and *S2P2* mutants was expected, as PsbA is a protein highly sensitive to photodamage [33,34,36]. However, the rate of PsbA degradation in *S2P2* mutants was significantly higher than in WT. Furthermore, PsbD and PsbC remained relatively stable in WT plants but were significantly degraded in the mutants.

The marked reduction in the levels of PsbD and PsbC in *S2P2* mutants, but not in wild-type plants, under severe light stress, combined with the accelerated degradation of PsbA, clearly indicates an increased intensity of degradation of these polypeptides in *S2P2* mutants (compared to WT), despite the obvious fact that protein levels always reflect a dynamic balance between synthesis and degradation [32,37,38]. These three polypeptides are typically present in thylakoid membranes in equimolar amounts in functional, dimeric PSII particles [31,32,37]. Thus, it is not particularly surprising that the changes in the levels of individual polypeptides observed in *S2P2* mutants are correlated with each other, despite the fact that the genes coding these polypeptides are located in the chloroplast genome within two different operons [39]. However, it is worth noting that the pattern of PsbA, PsbD, and PsbC polypeptide degradation observed in the *S2P2* mutants and the resulting unchanged stoichiometric ratio between these proteins is different from that observed in other *A. thaliana* mutants lacking the EGY1 or EGY2 intramembrane proteases [15,18,20]. In the case of the EGY1 and EGY2 mutants, we observed a disturbance in the stoichiometric ratios between PsbA and PsbD; namely, there was a clear overrepresentation of PsbA in relation to PsbD, which in itself was an unusual situation that was difficult to explain. Here, in the case of *S2P2* mutants, we observe a more classic situation in which a reduced amount of PsbA also corresponds to a reduced amount of PsbD. Considering that all chloroplast S2P mutants studied so far, namely EGY1, EGY2, and-now-*S2P2*, exhibit disturbances in the stoichiometry of PSII core proteins, one may suspect that there is some mechanism by which these proteases cooperate, ultimately maintaining normal levels of chloroplast-encoded PSII core polypeptides.

The altered stoichiometry of PSII core components under high light conditions can impair PSII function. Chlorophyll a fluorescence parameters confirmed that *S2P2* mutants exhibited increased sensitivity to photoinhibition. Specifically, a greater reduction in F_v_/F_m_ indicated severe damage to the PSII reaction center, while elevated NPQ values suggested enhanced thermal dissipation [40]. The observed increase in the F_0_ parameter in *S2P2* mutant lines likely reflects a decreased number of functional PSII centers, consistent with reduced levels of PSII core proteins. Consequently, these mutants displayed diminished electron transport efficiency (lower qP values) and heightened susceptibility to light-induced damage.

High irradiance also promotes the generation of reactive oxygen species (ROS), leading to oxidative stress and cellular damage. These highly reactive molecules are, in turn, the cause of oxidative stress and associated damage to many key proteins. ROS production is particularly prominent in chloroplasts and mitochondria [40,41,42]. The situation observed in the *S2P2* mutants, i.e., increased abnormalities in the content of key PSII core proteins and reduced PSII functionality, clearly seems to promote excessive ROS production in chloroplasts. The measurements of the content of superoxide radical and hydrogen peroxide confirm this assumption. ROS accumulation in *S2P2* chloroplasts was significantly higher than that recorded in WT plants. Simultaneously, we did not observe increased ROS production in *S2P2* mutants under non-stressful conditions. Once generated, the superoxide radical is quickly metabolized in chloroplasts to hydrogen peroxide. This is an extremely important element in the protection of the photosynthetic apparatus and, more broadly, chloroplasts and the entire cell in conditions such as light stress. The superoxide radical, if not scavenged, as an extremely reactive one, has a very large potential to destroy cellular components such as lipids, proteins, and DNA [43]. The enzyme playing a vital role in detoxifying superoxide radicals in chloroplasts is Cu/Zn superoxide dismutase 2 (CSD2), which is responsible for the dismutation of superoxide radicals into molecular oxygen and hydrogen peroxide [44,45]. CSD2 is crucial for plant survival and health, particularly under conditions that promote oxidative stress, such as high light intensity, drought, and nutrient deficiencies [45]. According to the literature information, CSD2 expression and activity are regulated by light and redox signals. In response to high irradiance and ROS production, the expression of CSD2 is upregulated to enhance its protective role [44]. In our study, CSD2 protein levels were more than twofold higher in *S2P2* mutants under high light compared to WT. As the product of CSD2 activity is hydrogen peroxide, its significantly higher level in *S2P2* mutants seems to be a consequence of the increased CSD2 content. The elevated level of CSD2 in mutant lines is an obvious response of *S2P2* mutants to the increased production of ROS under high light stress conditions.

The absence of chloroplast-localized *S2P2* protease results in the impaired accumulation of key PSII core proteins, compromised PSII efficiency, and increased vulnerability to high light-induced photoinhibition. This is accompanied by heightened ROS production and a robust induction of the antioxidant enzyme CSD2.

## 4. Materials and Methods

### 4.1. Plant Material, Growth, and Stress Conditions

*Arabidopsis thaliana* (L.) Heynh (ecotype Columbia) wild-type (WT) and two commercially available T-DNA insertion mutants, *s2p2–1* (SALK_046599C) and *s2p2–2* (SALK_071288), were used. Mutant lines were obtained from NASC (Nottingham Arabidopsis Stock Centre, Nottingham, United Kingdom). The homozygosity of both lines was previously confirmed with the PCR technique, and the lack of the *S2P2* protein was confirmed with the use of an anti-*S2P2* antibody [16].

All plants were grown on sphagnum peat moss and wood pulp in 42 mm Jiffy peat pellets (AgroWit, Przylep, Poland) under a 16 h light/8 h darkness photoperiod at an irradiance of 110 μmol m^−2^ s^−1^, relative humidity of 70%, and constant temperature of 22 °C for 4 weeks. For the light stress, the 4-week-old plants were exposed to continuous light at an intensity of 1000 mmol m^−2^ s^−1^ for 1, 3, 6., and 24 h. The light source was a white fluorescent light lamp, the Philips Master T-E-D 58 W/840 REFLEX Eco (Eindhoven, Holland).

### 4.2. S2P2 Gene Expression Analysis

Total RNA was extracted from *A. thaliana* WT leaves using the GeneMATRIX Universal RNA Purification Kit (EURX^®^, Gdańsk, Poland), following the manufacturer’s instructions. To remove genomic DNA contamination, the isolated RNA was treated with RNase-free DNase (Thermo Fisher Scientific, Waltham, MA, USA) according to the provided protocol. Complementary DNA (cDNA) synthesis was performed using 5 μg of total RNA with the RevertAid H Minus First Strand cDNA Synthesis Kit (Thermo Fisher Scientific, Waltham, MA, USA), employing random hexamer primers. cDNA synthesis was carried out for 60 min at 42 °C and was preceded by a 5 min incubation at 25 °C.

Quantitative real-time PCR (qRT-PCR) was conducted as described previously [16,19], utilizing the CFX96 Real-Time PCR Detection System (Bio-Rad, Hercules, CA, USA) and iTaq Universal SYBR Green Supermix (Bio-Rad, Hercules, CA, USA). Reactions were prepared in a final volume of 20 μL, including 1 μL of synthesized cDNA. The applied cycling conditions were as follows: 94 °C for 20 s, 55 °C for 30 s, and 68 °C for 30 s (40 cycles).

Relative expression of the *S2P2* gene under high light stress conditions was determined using the comparative CT (2^−ΔΔCT^) method [46]. For normalization, *CYP5* (At2g29960) was used as the endogenous control.

The primers used in this study were as follows:

*S2P2* (AT1G05140):

Forward: 5′-AGAACACAAGCTCTCGGTCG-3′

Reverse: 5′-ATCGGACCAAACCCTATCGC-3′

CYP5 (AT2G29960):

Forward: 5′-GAGAAAGGTGTAGGGAAGAGTGG-3′

Reverse: 5′-CAAACTTCTGACCATAGATTGATTC-3′

All primers were designed and validated by melting curve analysis to confirm specificity and the absence of primer-dimer formation using Primer3Input software (https://primer3.ut.ee/ accessed on 15 February 2023). Each sample was analyzed in three biological replicates, with three technical replicates per biological sample.

### 4.3. SDS-PAGE and Immunoblotting

SDS-PAGE was performed following the method of Laemmli [47], with minor modifications. Proteins were separated on 12% (*w*/*v*) polyacrylamide gels containing 6 M urea (Sigma-Aldrich, Loius, MO, USA). Gel running conditions were as follows: 25 mA per gel 90 min. Following electrophoresis, proteins were electrotransferred onto polyvinylidene fluoride (PVDF) membranes (Bio-Rad), and Western blotting was carried out as previously described by Adamiec et al. [19]. Before hybridization with antibodies, the membranes were transiently stained with Ponceu S to verify the transfer efficiency and control loading.

The PVDF membranes were blocked for 1 h with 4% BSA (BioShop, Burlington, Canada) and then incubated with primary antibodies for 90 min. This was followed by a 1 h incubation with secondary antibodies (Agrisera, Vännäs, Sweden), and signal development was achieved with a 5 min incubation using Clarity Western ECL Substrate (Bio-Rad). Protein bands were visualized with the ChemiDoc Imaging System (Bio-Rad).

Densitometric analysis of the immunostained bands was conducted using GelixOne software (Biostep GmbH, Burkhardtsdorf, Germany). Only blots demonstrating a linear relationship between signal intensity and the amount of protein loaded were included in the analysis. The linearity of signal detection was previously validated and reported by Adamiec et al. [19].

### 4.4. Antibodies

Custom polyclonal anti-*S2P2* antibodies were generated in rabbits by Davids Biotechnologie GmbH (Regensburg, Germany) using a highly specific synthetic peptide antigen (amino acid sequence: DNDPDSDIPVDDRNLLKNR). Commercial antibodies against PsbA, PsbD, PsbC, and CSD2, as well as the corresponding secondary antibodies, were obtained from Agrisera (Vännäs, Sweden).

### 4.5. Chlorophyll a Fluorescence Measurement

Chlorophyll *a* fluorescence measurements were performed using the FMS1 fluorometer (Photon System Instruments, Brno, Czech Republic), operated with Modfluor software. Prior to each measurement, leaves were dark-adapted for 30 min. The procedure followed the protocol established by Genty et al. [48].

The minimum fluorescence yield (F_0_) was recorded at the start of the measurement. The maximum quantum yield of PSII (F_v_/F_m_) were calculated according to Genty et al. [48]. Actinic light intensity matched the pre-dark adaptation irradiance: 110 μmol m^−2^ s^−1^ for control conditions and 1000 μmol m^−2^ s^−1^ for high light stress treatments.

Photochemical quenching (qP), and non-photochemical quenching (NPQ) were calculated following the formulas described by Maxwell and Johnson [49]. For each treatment variant, 30 individual plants were measured per replicate, with three biological replicates conducted in total.

### 4.6. Hydrogen Peroxide Detection

Hydrogen peroxide (H_2_O_2_) detection was carried out using two different methods, as described in our previous work [15].

DAB-Based Quantification: Quantitative determination of H_2_O_2_ levels in DAB-stained tissues was performed with modifications to the method of Boyidi et al. [50]. DAB-stained leaves were weighed, homogenized, and extracted in 2 mL of perchloric acid per 1 mg of fresh tissue. The homogenates were centrifuged at 10,000× *g* for 10 min, and absorbance was measured at 450 nm. H_2_O_2_ concentration was expressed in nmol per gram of fresh weight (FW).

Titanium-Based Spectrophotometric Assay: Spectrophotometric measurement of H_2_O_2_ was performed using the titanium (Ti^4+^) complex method, following Becana et al. [51]. *A. thaliana* leaves (0.25 g) were homogenized in 3 mL of 100 mM phosphate buffer (pH 7.8) with activated charcoal (1:5 ratio). The homogenate was centrifuged at 15,000× *g* for 30 min at 4 °C. For analysis, the supernatant was mixed in a cuvette with the titanium reagent (0.3 mM 4-(2-pyridylazo) resorcinol and 0.3 mM titanium potassium tartrate, mixed 1:1) and 100 mM phosphate buffer (pH 7.8). Absorbance was read at 508 nm and compared against a standard curve generated with known H_2_O_2_ concentrations (0–100 nmol). H_2_O_2_ levels were expressed as nmol per gram of fresh weight. The measurement was performed in 3 biological replicates and 3 technical replicates for each.

### 4.7. Detection of Superoxide Anion Radical

Superoxide radical (O_2_^•−^) accumulation was quantified based on the reduction in nitroblue tetrazolium (NBT), following the methodology described by Ratajczak et al. [52] and Doke [53]. A total of 0.5 g of leaf samples was incubated in 3 mL of 0.05 M potassium phosphate buffer (pH 7.8) containing 0.05% (*w*/*v*) NBT and 10 mM sodium azide. Incubation was carried out at room temperature in the dark for 30 min. Subsequently, the reaction mixture was subjected to heat treatment at 85 °C for 15 min, followed by immediate cooling on ice. The absorbance of the resulting solution was measured at 530 nm using a spectrophotometer. Superoxide radical generation was expressed as DA_530_/g FW leaves tissue. The measurement was performed in 3 biological replicates and 3 technical replicates for each.

### 4.8. Isolation of Leaf Proteins and Determination of Protein Concentration

Total soluble proteins were extracted from 100 mg of *A. thaliana* leaf tissue using a commercial protein extraction buffer (PEB; Vannas, Agrisera), following the manufacturer’s protocol. Protein concentration was quantified using a modified Lowry assay [54] with the DC Protein Assay kit (Bio-Rad, Hercules, CA, USA), according to the manufacturer’s instructions. Protein isolation was performed in 3 biological replicates and 3 technical replicates for each.

### 4.9. Statistical Analysis

Differences in the measured parameters were analyzed for statistical significance using one-way ANOVA. Means were regarded as significantly different at *p* ≤ 0.05.

## 5. Conclusions

Our results demonstrate that *S2P2* plays a critical role in maintaining the appropriate levels of three essential polypeptides within the PSII core complex—PsbA, PsbC, and PsbD. In *S2P2* mutant plants, the absence of *S2P2* resulted in a more pronounced decline in the abundance of these polypeptides under high light stress compared to wild-type controls. This disruption in PSII core stoichiometry was associated with a significant reduction in the maximum quantum efficiency of PSII (F_v_/F_m_) and an increase in the NPQ, indicating elevated photoinhibition and enhanced thermal energy dissipation in the mutants.

Furthermore, impaired PSII function in the *S2P2* mutants under high irradiance led to increased ROS accumulation, accompanied by a marked upregulation of CSD2, a key enzyme involved in the detoxification of superoxide radicals. Collectively, these findings suggest that *S2P2*, alongside other intramembrane S2P-type chloroplast proteases, contributes to the regulation of PSII core protein stoichiometry and efficiency, thereby modulating the plant’s sensitivity to photoinhibitory stress.

## Data Availability

All original contributions presented in this study are included within the article. For additional information or specific inquiries, readers are encouraged to contact the corresponding author.
